# Plant Cellulose as a Substrate for 3D Neural Stem Cell Culture

**DOI:** 10.3390/bioengineering10111309

**Published:** 2023-11-13

**Authors:** Lauren J. Couvrette, Krystal L. A. Walker, Tuan V. Bui, Andrew E. Pelling

**Affiliations:** 1 Department of Biology, University of Ottawa, Gendron Hall, 30 Marie Curie, Ottawa, ON K1N 5N5, Canada; 2 Department of Physics, University of Ottawa, STEM Complex, 150 Louis Pasteur Pvt., Ottawa, ON K1N 5N5, Canada

**Keywords:** NSC, 3D cell culture scaffold, biomaterial

## Abstract

Neural stem cell (NSC)-based therapies are at the forefront of regenerative medicine strategies for various neural defects and injuries such as stroke, traumatic brain injury, and spinal cord injury. For several clinical applications, NSC therapies require biocompatible scaffolds to support cell survival and to direct differentiation. Here, we investigate decellularized plant tissue as a novel scaffold for three-dimensional (3D), in vitro culture of NSCs. Plant cellulose scaffolds were shown to support the attachment and proliferation of adult rat hippocampal neural stem cells (NSCs). Further, NSCs differentiated on the cellulose scaffold had significant increases in their expression of neuron-specific beta-III tubulin and glial fibrillary acidic protein compared to 2D culture on a polystyrene plate, indicating that the scaffold may enhance the differentiation of NSCs towards astrocytic and neuronal lineages. Our findings suggest that plant-derived cellulose scaffolds have the potential to be used in neural tissue engineering and can be harnessed to direct the differentiation of NSCs.

## 1. Introduction

Neural stem cells (NSCs) are self-renewing cells that proliferate in vitro and maintain the capacity to differentiate into neurons, astrocytes, and oligodendrocytes [1]. NSC transplantation is being investigated as a therapeutic strategy for numerous disorders of the central nervous system [2,3], and many preclinical studies report promising results [4,5]. However, there are currently important limitations to the efficacy of NSC therapies such as low transplant survival and poor efficiency of neuronal differentiation. To overcome these issues, biocompatible scaffolds have emerged as a vehicle to engraft NSCs while supporting the survival of the transplanted cells [6]. Cell scaffolds can enhance the therapeutic efficacy of NSCs by promoting strong cell adhesion, guiding cell migration, and shielding transplanted NSCs from the cytotoxic injury environment occurring in CNS injuries [6,7]. Moreover, since NSC differentiation is mechanosensitive [8,9], the physical properties of scaffolds such as porosity and elastic modulus can be exploited to influence stem cell differentiation [10,11,12]. It has been shown that stiffer matrices tend to be myogenic, whereas softer matrices favor neuronal differentiation [13] and promote expression of the neuronal marker βIII-tubulin in adult neural stem cells [14]. Scaffold alignment also guides stem cell differentiation [15,16], and studies indicate that anisotropic topographies support further axonal extension relative to isotropic topographies [17].

In addition to physical cues, manipulating surface chemistry has been extensively investigated as a method to direct migration, proliferation, and differentiation of NSCs [18,19,20,21]. For instance, Ge et al. studied the effects of Poly-L-ornithine (PLO) on cell behavior in vitro and determined that NSCs cultured on PLO had enhanced cell migration [22]. We hypothesized that this coating would enhance the attachment and growth of cells to a plant scaffold and encourage migration into its channels. In addition, PLO was shown to induce preferential differentiation into neuronal and oligodendrocytic cell types [23]. This is of particular importance, since one of the key challenges in NSC therapies is directing the differentiation of neural stem cells towards the neuronal lineage. Finally, Ping, Jie et al. demonstrated that poly-l-ornithine enhances myelin regeneration and promotes locomotor recovery in an animal model of focal demyelination [24], suggesting that PLO has potential to be part of a regenerative therapeutic strategy for injuries of the central nervous system. Likewise, NH_2_-terminated surfaces have been shown to promote neuronal differentiation of NSCs [20]. Further, some researchers have harnessed both topography and surface chemistry to direct stem cell behavior on scaffolds [25,26].

In recent years, various three-dimensional cell culturing methods have been developed using scaffold-based technologies. These systems more accurately represent the in vivo microenvironment compared to traditional two-dimensional culture on polystyrene [27], and the behavior of cells within 3D systems is more physiologically relevant, making them better cell culturing methods for tissue engineering and drug discovery [28,29,30,31,32,33]. As such, various scaffold-based neural constructs have been developed for use in disease modeling, regenerative medicine, and the study of the stem cell niche [34,35,36,37].

In the past decade, plant-derived scaffolds have been used successfully in numerous independent studies by many research groups worldwide [38,39,40,41,42,43,44,45,46,47,48,49,50,51,52,53,54,55]. The biocompatibility of plant scaffolds has been extensively studied both in vitro and in vivo. Many plant species and tissues such as apple [56], leek [41], parsley [43], spinach [46], green onion [47], *Flammulina velutipes* [54], tobacco BY-2 cells [55], *Camellia japonica* [53], and brown seaweed [57] have been studied with many cell lines including C2C12 myoblasts, human foreskin fibroblasts, HeLa cells [58], NIH 3T3 cells [51], human mesenchymal stem cells, and human pluripotent stem cell-derived cardiomyocytes [43]. For example, spinach leaves were decellularized while maintaining their vascular architecture, which was then repopulated with human dermal microvascular endothelial cells [49]. In addition to the extensive in vitro biocompatibility testing, several plant species have been studied in vivo for applications such as cardiac tissue repair [59], tissue engineering of skin [49,60,61], tendons [40], and bone [40,50,62,63,64]. Plant-based biomaterials were shown to support cell infiltration and vascularization in vivo [39,56,58]. In one study, a *Flammulina velutipes* mushroom was successfully used as a nerve guidance conduit in a rat model of sciatic nerve defect [54]. As evidenced above, plant-derived biomaterials are becoming increasingly attractive for biomedical applications, which can be attributed in part to improved cost effectiveness, scalability, and lower immunogenicity relative to animal sources.

Here, we investigate the viability of a plant-derived biomaterial as a 3D, in vitro culture system for adult rat neural stem cells. We hypothesized that the stalks of *Asparagus officinalis*, which are composed of linearly arranged microchannels, would produce a scaffold with an interesting surface topology for the culture of neural stem cells, as topology is known to influence NSC differentiation and axon guidance. We first examined the physical characteristics of the asparagus scaffold with scanning electron microscopy and mechanical testing. Next, we assessed the ability of the scaffold to support the attachment and migration of NSCs for various time periods. Further, we examined the differentiation potential of neural stem cells in this 3D culture system by immunostaining for markers including neuron-specific β-III tubulin and glial fibrillary acidic protein.

## 2. Methods

### 2.1. Biomaterial Production

Asparagus scaffolds were prepared utilizing decellularization methods described previously [39,51]. Using a 4 mm biopsy punch, Asparagus officinalis sections were cut and placed into a 50 mL Falcon tube containing 0.1% sodium dodecyl sulphate (SDS) (Sigma-Aldrich, Markham, ON, Canada). The dimensions of the final scaffold were 4 mm in diameter and 1.2 mm in thickness. Samples were shaken for 72 h at 180 RPM at room temperature. The resulting cellulose scaffolds were then transferred into new sterile microcentrifuge tubes, washed, and incubated for 12 h in phosphate-buffered saline (PBS). Following the PBS washing steps, the asparagus were then incubated in 100 mM CaCl_2_ for 24 h at room temperature and washed 3 times with dH_2_O. Samples were then sterilized in 70% ethanol overnight. Finally, they were washed 12 times with sterile 1X PBS.

### 2.2. Scanning Electron Microscopy

Scanning electron microscopy was performed at the 2-week timepoint. NSC-seeded scaffolds were fixed with 4% PFA and dehydrated through successive gradients of ethanol (50%, 70%, 95%, and 100%). Samples were dried with a critical point dryer (SAMDRI-PVT-3D, Tousimis, MD, USA) then gold-coated at a current of 15 mA for 3 min with a Hitachi E-1010 ion sputter device. SEM imaging was conducted at voltages ranging from 2.00 to 10.0 kV on a JSM-7500F Field Emission SEM (JEOL, Peabody, MA, USA).

### 2.3. Mechanical Testing

After 7 days in culture media at 37 °C, scaffolds (4 mm diameter × 1.2 mm height) were placed onto a CellScale UniVert (CellScale, Waterloo, ON, Canada) compression platform for tensile testing. Each scaffold (n = 15) was compressed mechanically to a maximum 30% strain, at a compression speed of 50 µm/s. The elastic modulus was determined from the slope of the linear region of the resulting stress–strain curves.

### 2.4. Cell Culture, Scaffold Seeding and Differentiation

The resulting cellulose scaffolds were incubated in poly-L-ornithine (Sigma, Canada, 20 μg/mL in dH_2_O) overnight at room temperature. PLO-coated scaffolds were rinsed twice with sterile water before being transferred into a 96-well plate. Rat adult hippocampal neural stem cells (SCR022, Sigma, Canada) were cultured in serum-free medium (1X KnockOut D-MEM/F-12 with 2% StemPro Neural Supplement (A1050801 ThermoFisher, Toronto, ON, Canada), 20 ng/mL bFGF, 20 ng/mL EGF, and 2 mM GlutaMAX-I) and incubated at 37 °C and 5% CO_2_. Culture vessels were also coated with 20 μg/mL poly-L-ornithine and 10 μg/mL laminin, as described above. An 80 μL droplet containing 200,000 cells was deposited onto every scaffold, which was then incubated at 37 °C and 5% CO_2_. After 4 h of incubation, 2 mL of StemPro NSC SFM complete medium was added to each scaffold, which was then incubated for 48 h before the scaffolds were transferred to a new 96-well plate. For 2–4 weeks, the medium was exchanged daily. For neuronal and astrocyte differentiation, cells were cultured in 1X KnockOut DMEM/F-12 supplemented with 2% B27 (17504044, ThermoFisher, Canada), 2 mM GlutaMAX-I (ThermoFisher, Canada) for 7 days.

### 2.5. Staining and Confocal Microscopy

Cell attachment and morphology was documented using phase contrast microscopy at day 3, 7, and 14 post seeding. Staining and confocal microscopy was performed at 3 and 14 days in culture. NSC-seeded scaffolds were fixed with warm 4% paraformaldehyde for 10 min then incubated for 3 min in warm permeabilization buffer (0.5% Triton-X, 20 mM HEPES, 300 mM Sucrose, 50 mM NaCl, 3 mM MgCl_2_, 0.05% sodium azide). Samples were then incubated for 15 min in fluorescein phalloidin (1:100, F432, ThermoFisher, Canada) to stain F-actin. Samples were rinsed with PBS and incubated in Hoechst (1:200, ThermoFisher, Canada) for 10 min to label nuclei. Scaffolds were incubated in 0.2% Congo red (Sigma, Canada) for 15 min before a final PBS rinse.

For immunostaining GFAP and β-tubulin, cells or cell-seeded scaffolds were fixed in 4% paraformaldehyde for 15 min at room temperature. Samples were incubated for 5 min in permeabilization buffer (0.5% Triton-X, 20 mM HEPES, 300 mM Sucrose, 50 mM NaCl, 3 mM MgCl_2_, 0.05% sodium azide). After blocking in 6% Normal Goat Serum in 1X PBS for 10 min, samples were incubated in rabbit anti-GFAP antibody (AB5804 Sigma, Canada, 1:1000 in 1X PBS) or mouse anti-β-III tubulin antibody (MAB1195 R&D systems, Toronto, ON, Canada, 10 ug/mL in 1X PBS) overnight at 4 °C. The following day, samples were washed twice in 1xPBS (5 min, RT), followed by a 2 h incubation in secondary antibody: goat anti-rabbit IgG Alexa Fluor 488 (A11008 Invitrogen, Burlington, ON, Canada, 1:500 in 1X PBS) or goat anti-mouse IgG Alexa Fluor 594 (A11005 Invitrogen, Canada 1:200 in 1X PBS). Samples were then washed in 1X PBS and counterstained with Hoescht 33342 (1:2000 in 1X PBS) for 10 min.

All samples were then mounted in Vectashield (Vector Labs, Newark, CA, USA) and imaged on with a Nikon A1R laser scanning confocal microscope (Nikon, Mississauga, ON, Canada) with appropriate filter sets and laser lines.

### 2.6. Image Analysis

For ßIII-tubulin stains, analysis was performed on 13 images at 40X magnification for each condition (2D and 3D). Total cell number was determined using cell counting Hoescht-labeled nuclei using FIJI (National Institutes of Health, Bethesda, MD, USA). Cells were considered ßIII-tubulin^+^ when signals from red and blue channels were colocalized. For GFAP stains, analysis was performed on 6 images at 40X magnification for each condition (2D and 3D). Cells were considered GFAP^+^ when signals from green and blue channels were colocalized.

### 2.7. Alamar Blue Cell Proliferation Assay

To measure cell proliferation, an Alamar Blue assay was performed according to the manufacturer’s protocol. Briefly, PLO-coated asparagus scaffolds were placed into the wells of a 96-well plate and seeded with 100,000 adult rat hippocampal neural stem cells. After 1, 2, or 5 days, cell-seeded scaffolds were transferred into a new well, and 200 µL of fresh media containing 10% Alamar Blue (cat. BUF012A, Bio-Rad, Toronto, ON, Canada) was added to each scaffold. After 4 h of incubation at 37 °C, 100 µL of media was removed from each well and deposited into an empty well before reading absorbance at 570 nm and 600 nm with a spectrophotometer (Epoch 2, BioTek, Winooski, VT, USA). Each timepoint included biological triplicates for cell-seeded scaffolds (n = 3), 2D controls (NSCs grown on a PLO-coated well, n = 3), and blanks (media only, n = 3). Absorbances were corrected by subtracting the average absorbance of blanks at 570 nm and 600 nm.

### 2.8. Statistical Analysis

*p* values were calculated using two-tailed Student’s *t*-test. Results were considered as statistically significant when *p* < 0.01. Numerical data are expressed as mean ± standard deviation.

## 3. Results

### 3.1. Characterization of Plant Cellulose Scaffold

**Figure 1:**
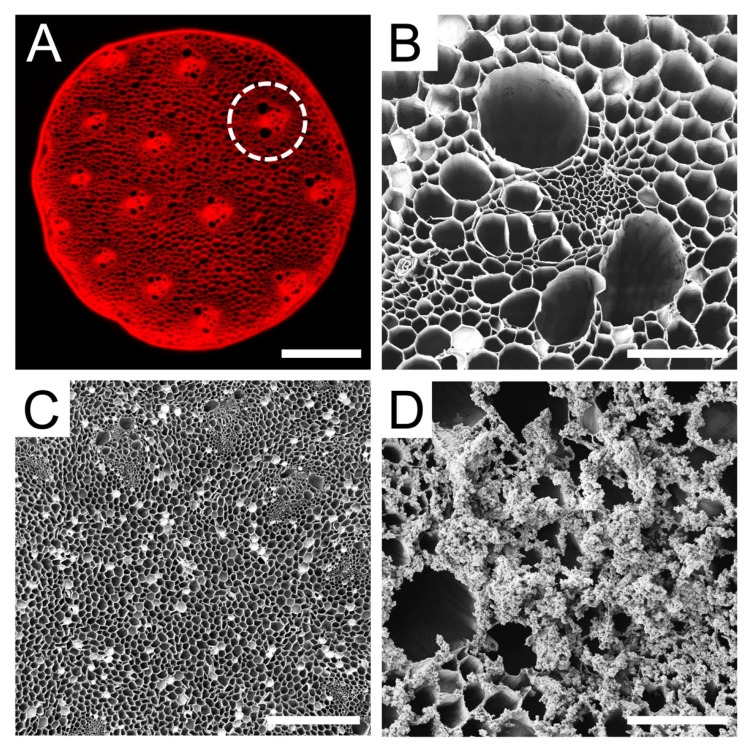
Cross sections of decellularized *Asparagus officinalis* scaffolds. (**A**) Confocal microscope maximum intensity projection of a decellularized asparagus scaffold stained with Congo red for cellulose. The decellularization process preserves vascular bundles (VBs, circled), which are microchannels that run along the asparagus stalk and are separated by porous parenchyma tissue (scale bar = 1 mm) (**B**) High magnification SEM of an individual VB within scaffold which reveals the long microchannels (scale bar = 100 µm). (**C**) SEM of the parenchyma tissue which reveals numerous pores with a wide distribution of diameters (scale bar = 500 µm). (**D**) Higher magnification SEM of a scaffold coated with poly-L-ornithine, a positively charged synthetic amino acid which gives the scaffold surface a sabulous appearance (scale bar = 100 µm). Such appearance is never observed on uncoated, decellularized scaffolds and only appears after PLO coating.

Raw *Asparagus officinalis* stalks were cut into discs of 4 mm in diameter and 1.2 mm in height, which were then decellularized to remove all native cells. The resulting scaffold was composed of vascular bundles (VBs) interspersed between parenchyma (Figure 1A). Naturally occurring structures within the scaffold were characterized using scanning electron microscopy (Figure 1B–D). A majority of the surface of the scaffold consisted of parenchyma that had an average pore diameter of 39 ± 15 µm. In addition, each scaffold was found to contain 14 ± 2 vascular bundles, which are aligned channels that traverse the length of the scaffold, with an average spacing of 602 ± 61 µm.

The distribution of channel diameters observed in these scaffolds may be conducive to cell survival, since such porous networks have previously been demonstrated to enable nutrient exchange and waste removal within scaffolds [65,66]. The Young’s modulus of the cellulose scaffold in culture media at 37 °C is 128 ± 20 kPa (n = 5) when measured parallel to the long axis. Moreover, the scaffolds were coated overnight in poly-L-ornithine (PLO) to promote cell attachment. Surface modification with PLO was confirmed with SEM (Figure 1D).

### 3.2. Cellulose Scaffold Supports Rat NSC Attachment and Proliferation

**Figure 2:**
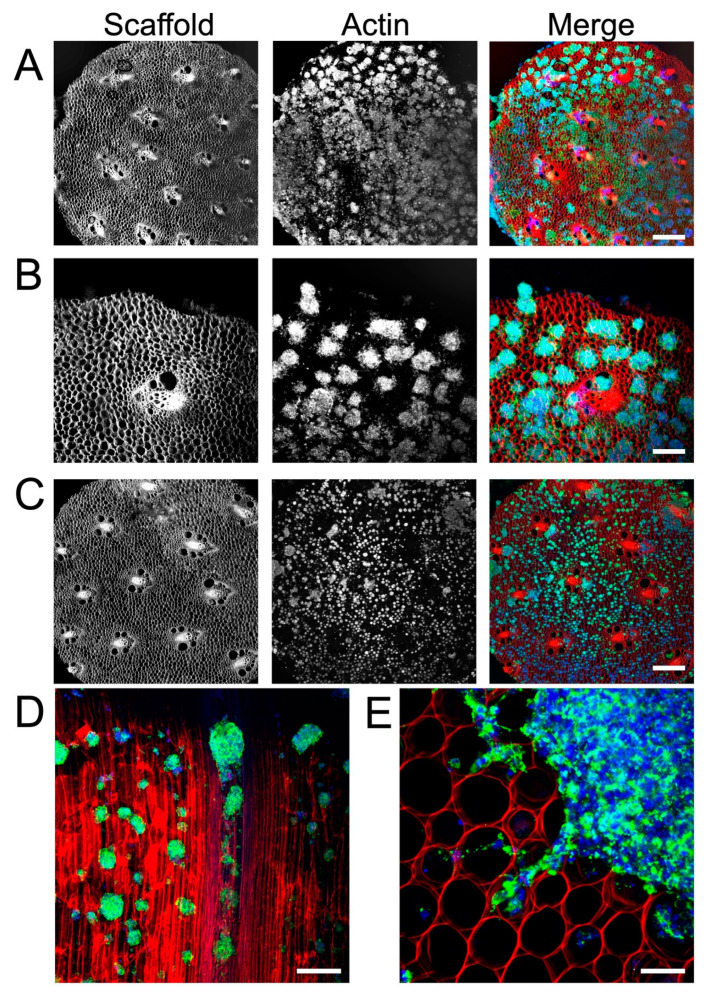
Confocal microscopy (maximum intensity projection) of adult rat NSCs cultured on decellularized, PLO-coated asparagus scaffold. F-actin was stained with phalloidin (green), nuclei were stained with Hoechst 33342 (blue), and the cellulose scaffold was stained with Congo red (red). (**A**) A 4X magnification image of NSCs grown on scaffold for 3 days revealing numerous neurospheres (scale bar = 500 µm). (**B**) A 10X magnification cross section of NSCs on scaffold at 3 days in culture shows the distribution of neurospheres at greater detail (scale bar = 100 µm). (**C**) A 4X magnification of NSCs grown on scaffold for 14 days reveals the continued presence and distribution of neurospheres on the scaffold surface (scale bar = 500 µm). (**D**) After sectioning the scaffold longitudinally along its long axis (parallel to the direction of the VB microchannels), a 10X magnification image reveals the NSCs migrating into scaffold channels at 3 days in culture (scale bar = 200 µm). The highly aligned structure of the cellulose scaffold is easily observed (red) and the presence of NSCs and neurospheres are observed deep within the scaffold. (**E**) A 40X magnification cross section of NSCs grown on scaffold for 14 days (scale bar = 50 µm) reveals groups of NSCs migrating and projecting out of the edge of a single neurosphere.

**Figure 3:**
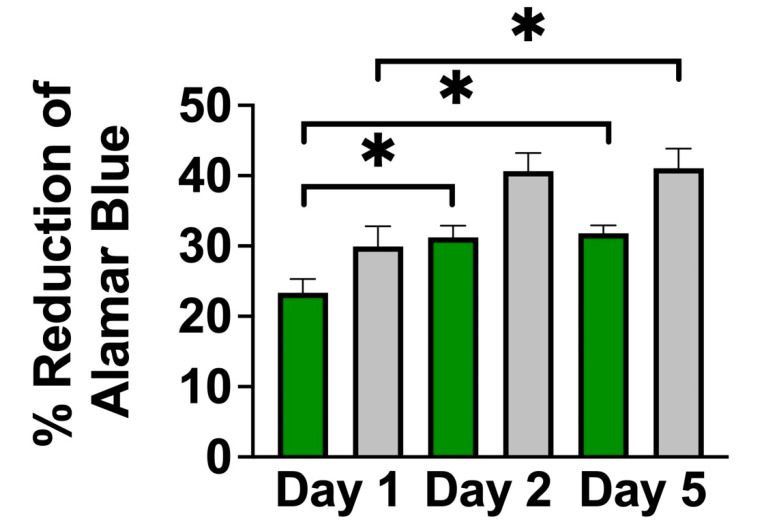
Evaluation of cell proliferation with Alamar Blue assay. The percent reduction of Alamar Blue reagent by NSCs grown on a cellulose scaffold (in green) compared to a polystyrene culture plate (in grey) over 5 days in culture. Metabolic activity of cells increased over 5 days in culture, indicating continued growth of NSCs on the scaffold. Statistical significance (* indicates *p* < 0.01) was determined using a student’s *t*-test. (Error bars represent standard deviation, N = 3 for each condition).

A single-cell suspension of NSCs was seeded onto PLO-coated scaffolds and cultured for 3 to 14 days. As early as 3 days in culture, neurospheres had attached to the cellulose scaffold and were visualized using F-actin staining (Figure 2A). Many neurospheres with diameters of 50 µm to 300 µm were found on the surface of the scaffold (Figure 2B). By 14 days in culture, the same high density of neurospheres on the scaffolds was observed to persist (Figure 2C). In order to examine how the NSCs penetrated into the VB microchannels, the scaffold was sectioned longitudinally along its long axis. This allowed us to visualize the migration of NSCs and neurospheres down the cellulose channels (Figure 2D). Importantly, NSC cells and neurospheres were observed inside of the scaffold as well as on its surface. Finally, we also observed that NSCs were able to migrate out of the attached neurospheres (Figure 2E). Groups of cells were observed migrating and extending out from several neurospheres onto the scaffold. In addition to microscopic examination, cell proliferation was also assessed. This was achieved on the scaffolds with an Alamar Blue assay, which monitors the chemical reduction of culture media to detect metabolic activity of cells. Over 5 days in culture (Figure 3), the percentage of reduced Alamar Blue reagent increased progressively, indicating the continued growth of NSCs on the scaffold. When compared to NSCs grown as a monolayer on a polystyrene culture plate, cells on the scaffold had a slight reduction in metabolic activity during the first 5 days of growth but exhibited a similar trend.

### 3.3. Plant Cellulose Scaffold Enhances Neuronal and Astrocytic Differentiation

**Figure 4:**
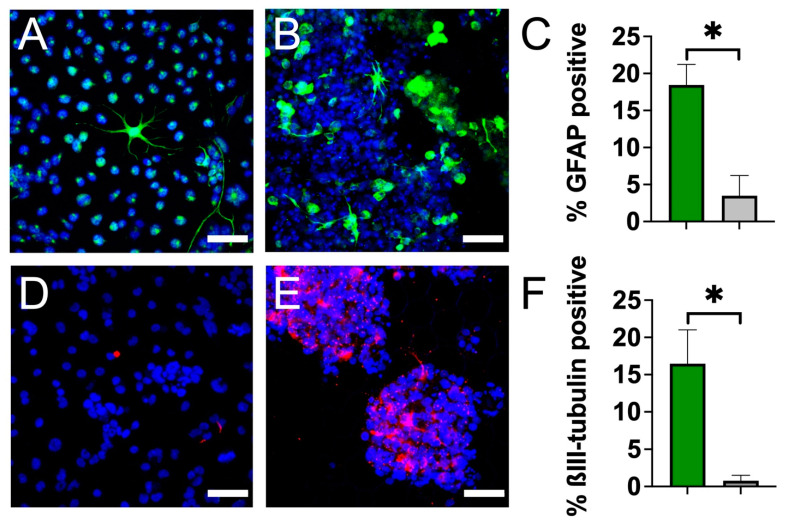
NSC lineage analysis with immunostaining reveals enhanced neuronal and astrocytic differentiation on cellulose scaffold. Representative confocal microscopy (maximum intensity projections) of adult rat NSCs after 7 days in culture in differentiation media. Nuclei were stained with Hoechst 33342 (blue). (**A**) NSCs grown on PLO-coated culture plates (2D) stained for GFAP (green) (scale bar = 50 µm). GFAP-positive cells were identified as possessing green signal throughout the entire cell body and not just localized to the nucleus. (**B**) NSCs grown on PLO-coated scaffold (3D) stained for GFAP (green) (scale bar = 50 µm). (**C**) Percentage of GFAP-positive adult rat NSCs after 7 days in culture on 3D scaffolds (in green) compared to 2D polystyrene plates (in grey). Statistical significance (* indicates *p* < 0.01) was determined using a student’s *t*-test. (**D**) NSCs grown on PLO-coated culture plates (2D) stained for ßIII-tubulin (red) (scale bar = 50 µm). (**E**) NSCs grown on PLO-coated scaffold (3D) stained for ßIII-tubulin (red) (scale bar = 50 µm). (**F**) Percentage of ßIII-tubulin-positive adult rat NSCs after 7 days in culture on 3D cellulose scaffold (in green) compared to 2D polystyrene plate (in grey). Statistical significance (* indicates *p* < 0.01) was determined using a student’s *t*-test.

To determine the effects of this 3D culture system on NSC differentiation potency, we monitored the expression of lineage-specific markers. A single-cell suspension of NSCs was simultaneously seeded onto PLO-coated scaffolds and onto a PLO-coated culture plate at equal seeding densities. After seven days in the differentiation media, the cells were fixed and immunostained for glial fibrillary acidic protein (GFAP, an astrocytic marker) or neuron-specific ßIII-tubulin (immature neuron marker). Immunostaining revealed a significantly higher fraction of GFAP-positive cells were observed on the cellulose scaffold (18.45 ± 2.8%) compared to the 2D monolayer on a polystyrene culture plate (3.50 ± 2.7%) (Figure 4A–C) (*p* < 0.01, n = 6). Similarly, immunostaining revealed an enhanced expression of ßIII-tubulin on the 3D scaffold (Figure 4D–F). Cells in a 2D condition were 0.79 ± 0.7% ßIII-tubulin positive, whereas cells grown on the 3D scaffold were 16.46 ± 4.5% ßIII-tubulin positive, which represents a significant increase in the expression of this early neuronal marker (*p* < 0.001, n = 13). Both findings demonstrate that NSCs retain their ability to differentiate into various lineages when cultured in this 3D cellulose scaffold.

## 4. Discussion

There is growing interest in the use of plant-based scaffolds in tissue engineering [38,41,42]. Importantly, since they are composed primarily of cellulose, plant scaffolds are biocompatible and are therefore ideal for applications such as mammalian cell culture or tissue engineering. In this study, we developed a 3D cell culture scaffold consisting of decellularized *Asparagus officinalis* stalks that supported the attachment, proliferation, and differentiation of rat adult neural stem cells. After decellularization, the cellulose scaffold was seeded with primary neural stem cells isolated from the hippocampus of adult Fisher 344 rats. Microscopic examination of the scaffolds revealed a system of aligned channels with various diameters, which we predicted would allow for efficient transport of nutrients and provide guidance cues for cell attachment. Other groups have demonstrated that the topography of scaffolds affects cell migration and alignment. In particular, many report that cells respond to parallel linear structures by aligning along the axis of the substrate [17,67]. We believe the aligned channels within our plant scaffold can potentially act as physical scaffolding to guide the alignment and migration of NSCs by providing cues for contact guidance. Within the scaffold, the parenchyma has pores with smaller diameters (~39 µm) relative to the pores of the vascular bundles. However, others have reported successful 3D culture of NSCs in scaffolds with pore sizes that are in a similar size range [68,69]. Our results demonstrate that within 3 days, NSCs attached to the scaffold, both as individual cells and neurospheres of diverse sizes (Figure 2). Groups of NSCs appear to migrate out from the neurospheres attached to PLO-coated scaffolds. This behavior can be explained by the fact that PLO enhances migration by promoting filopodia formation [22]. In fact, Poly-L-ornithine is widely used in neuroscience to enhance cell attachment and promote cell migration. Subsequent F-actin staining revealed the morphology of neurospheres within the biomaterial and highlighted the migration of NSCs into the channels of the scaffold. Further, NSCs were found to proliferate within the 3D culture system, as demonstrated with an Alamar Blue assay (Figure 3). Taken together, these data suggest the scaffold is biocompatible and has appropriate physical characteristics to allow for neural stem cell growth in vitro.

The behavior of cells cultured in 3D systems is often different from that of those cultured in 2D systems, including the rate of proliferation [70,71]. Interestingly, the percent reduction in Alamar Blue reagent was consistently lower in the 3D scaffold compared to the 2D monolayer culture system, suggesting a slight inhibition of NSC growth on the scaffolds. This difference can be attributed in part to the increased heterogeneity in the 3D culture system, where NSCs assembled into neurospheres attached to the scaffold. While the outer surface of these neurospheres consists of cells with high rates of proliferation, the inner layers of NSCs tend to be quiescent or necrotic due to reduced access to oxygen, nutrients, and growth factors. Therefore, this difference in reduction of Alamar Blue may result from heterogeneity rather than purely from differences in the rate proliferation.

Finally, NSCs grown on the scaffolds were cultured in differentiation media, and their expression of cytosolic markers GFAP and ßIII-tubulin was evaluated after 7 days. Remarkably, this plant-derived scaffold appears to enhance NSC differentiation towards astrocytes and neurons, as evidenced by significant increases in GFAP-positive and ßIII-tubulin-positive cells on the scaffold compared to 2D controls (Figure 4). It is well established that the differentiation of stem cells can be influenced by several extrinsic and intrinsic factors including chemical cues, the physical characteristics of the environment, and the surrounding cell types [15,16,17]. Therefore, the differences observed in our differentiation experiments are likely due to a complex interplay of cues provided by the scaffold. An important factor in guiding the fate of NSCs is scaffold architecture and anisotropy. It was previously reported that anisotropic contact promotes neuronal differentiation of NSCs [72]. In accordance, we propose that the aligned channels of the plant scaffold provide geometric cues that may modulate differentiation toward higher neurogenesis. In addition, scaffold elasticity is another key factor which mediates stem cell fate [34]. To determine the elastic modulus of the scaffold, mechanical tests were performed after 1 week in media at 37˚C. The Young’s modulus of the scaffold was determined to be 128 ± 20 kPa when measured parallel to the long axis, which is softer than polystyrene cell culture plates (E = 3.73 GPa) [73]. The elasticity of the scaffold is within the range reported for brain and spinal cord tissue [74,75] and is similar to other scaffolds that are currently being investigated for therapeutic use in spinal cord injury (260–300 kPa) [76]. Previous studies have shown that softer growth substrates tend to favor neuronal differentiation [13,14,77]. Here, the difference in stiffness between the scaffold and the polystyrene cell culture plate likely contributed to the increase in neuronal differentiation. In addition, the 3D growth environment may have enhanced cell–cell signaling for lineage differentiation, which could induce more differentiation of NSCs on the scaffold relative to 2D culture, as others have reported [78,79,80].

Interestingly, we observed many neurospheres forming in the confined circular pores of the parenchyma tissue (Figure 2A–C). In previous work, we have shown how embryonic stem cell spheroid formation can be stimulated by the physical confinement of cells in small microscale environments [81]. This is not dissimilar to what takes place in traditional hanging drop and round bottom multi-well culture models designed for stimulating spheroid formation [82]. In such environments, cells become trapped and interact more frequently, which nucleates the formation of a spheroid. Here, we speculate that a similar phenomenon is taking place, leading to a significant increase in the number of spheroids on and inside of the scaffold as opposed to on a flat substrate. However, at this point, the precise mechanism which leads to enhanced NSC differentiation on these scaffolds is unclear, and future work will be required to understand this phenomenon. In summary, we have demonstrated that cellulose scaffolds support the growth and differentiation of neural stem cells in vitro. Our findings suggest that plant-derived scaffolds could facilitate the production of large numbers of specifically differentiated cells needed for NSC research or regenerative medicine.

## Data Availability

The data presented in this study are available on request from the corresponding author.
